# Nutrition Incentives Associated With Improved Outcomes: 2020–2023 Results From the U.S Gus Schumacher Nutrition Incentive Program

**DOI:** 10.1016/j.focus.2025.100348

**Published:** 2025-04-11

**Authors:** Carmen Byker Shanks, Whitney Fung Uy, Nanhua Zhang, Courtney A. Parks, Hollyanne E. Fricke, Kenneth Resnicow, Nadine Budd Nugent, Amy L. Yaroch

**Affiliations:** 1 Center for Nutrition & Health Impact, Omaha, Nebraska; 2 Cincinatti Children’s Hospital Medical Center, University of Cincinnati College of Medicine, Cincinnati, Ohio; 3 University of Michigan School of Public Health, Ann Arbor, Michigan

**Keywords:** Nutrition incentives, public health, intervention, food security, fruits and vegetables, dietary intake

## Abstract

**Introduction:**

Populations with low income and food insecurity report low fruit and vegetable intake and high associated diet-related disease. The Gus Schumacher Nutrition Incentive Program supports grantees to implement nutrition incentive projects nationwide to provide individuals participating in the Supplemental Nutrition Assistance Program with incentives to facilitate the purchase and consumption of fruits and vegetables. This study examines the associations between the length of Gus Schumacher Nutrition Incentive Program nutrition incentive participation and fruit and vegetable intake, food insecurity, and perceived health status.

**Study design:**

Nutrition incentives were available to eligible participants during grantees’ multiyear award period between 2020 and 2023. Sixty-five Gus Schumacher Nutrition Incentive Program grantees collected cross-sectional surveys from a convenience sample of nutrition incentive project participants.

**Settings/participants:**

A total of 23,736 adult Supplemental Nutrition Assistance Program recipients (aged ≥18 years) who participated in a nutrition incentive project and completed the cross-sectional survey were involved.

**Intervention:**

Nutrition incentive projects varied in implementation at grocery stores, farmers markets, and other food retail spanning across all regions of the U.S.

**Main outcome measures:**

Main outcome measures included fruit and vegetable intake, food insecurity, and perceived health status by length of participation.

**Results:**

Participants in nutrition incentives at >6 months (cups per day=2.91) demonstrated significantly higher fruit and vegetable intake than first-time participants (cups per day=2.73) and those ≤6 months (cups per day=2.76). Significance (*p*<0.05) was observed for reduced food insecurity (≤6 months versus first time [OR=0.66, 95% CI=0.60, 0.73], >6 months versus first time [OR=0.60, 95% CI=0.55, 0.65]) and better perceived health (≤6 months versus first time [OR=1.28, 95% CI=1.17, 1.41], >6 months versus first time [OR=1.48, 95% CI=1.35, 1.62]). Differences by racial and ethnic groups as well as age existed for all main outcomes.

**Conclusions:**

Nutrition incentives appear to increase fruit and vegetable intake, reduce food insecurity, and result in better perceived health. They are a meaningful public health intervention to address key differences in diet-related outcomes.

## HIGHLIGHTS

• Longer participation in nutrition incentives is linked to increased fruit and vegetable intake.
• Increased length of participation in nutrition incentives decreased food insecurity odds.
• Longer nutrition incentive participation improved the odds of better perceived health.
• Nutrition incentive outcomes vary by race, ethnicity, and age group.

## Graphical Abstract

**Figure:**
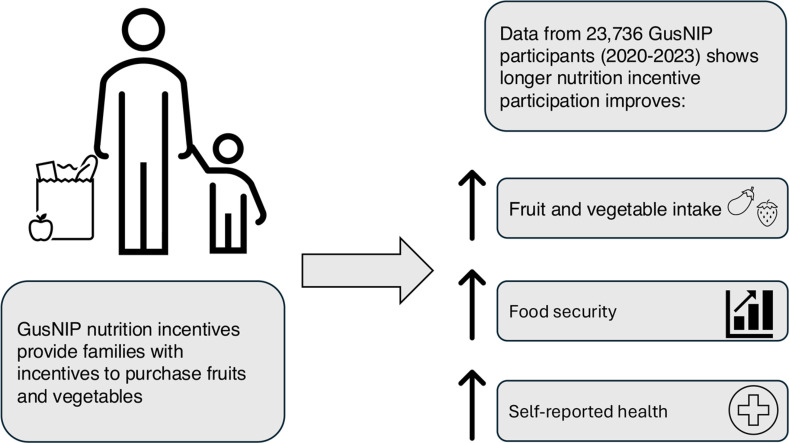

## INTRODUCTION

The proportion of adults in the U.S. who meet fruit and vegetable (FV) dietary recommendations remains low (12.3% and 10.0%, respectively).^1^^,^^2^ Moreover, populations with low income and food insecurity report challenges in achieving recommended FV intake (FVI).^2^, ^3^, ^4^, ^5^, ^6^, ^7^, ^8^ Food insecurity, or limited access to enough food for an active and healthy life, is associated with diet-related disease.^9^ Affordability of healthy foods, including FVs, is a significant driver of dietary choice among populations with low income and those experiencing food insecurity.^8^^,^^10^

Nutrition incentives have the potential to improve affordability of FVs and decrease food insecurity in U.S. populations with low income. In general, nutrition incentives provide individuals participating in the Supplemental Nutrition Assistance Program (SNAP) with incentives to reduce costs and increase purchases and consumption of FVs. To demonstrate, a nutrition incentive (NI) participant might receive a 1:1 incentive, where they get 1 dollar to purchase additional FVs for every 1 dollar of FVs purchased using SNAP Electronic Benefit Transfer (EBT) at participating food retailers. That is, an NI participant who spends $10 of SNAP on FVs would be able to get $20 worth of produce. It is important to note that there is variability in both the incentive amounts and the types of food retailers involved across different projects that distribute NIs. In addition, although produce prescriptions are another form of healthy food incentive, they are not addressed in this paper.

Varied results from studies with heterogenous methodologic designs have led to gaps in understanding of the NI projects on participant outcomes. Longitudinal studies about single NI projects have found that NI participation is associated with higher FVI.^11^, ^12^, ^13^ Conversely, other research demonstrates that NI projects did not increase FVI after participation.^14^, ^15^, ^16^ Across all NI research, the projects were implemented at various scales (e.g., single store, county, city, state) across different food retail settings (e.g., grocery stores, farmers markets, corner stores), and with varying incentive models (e.g., 1:1 or 2:1 match), suggesting that the most effective implementation approaches are yet to be determined.

Microsimulation research has suggested that scaling up NI could result in positive outcomes, with FV incentives linked to increased purchasing, a reduction in diet-related chronic diseases, improvements in quality-adjusted life years, and a positive economic impact.^17^, ^18^, ^19^ However, likely owing to the heterogeneity of NI projects across the country and implementation research to date being limited in scope, these effects have not been consistently observed.

Previous studies found mixed results for the effects of NI on food insecurity. A study at 17 farmers markets in Utah found that food insecurity decreased after NI project participation.^12^ Furthermore, a study comparing $20 with $10 match incentive models found that higher match amounts for SNAP participants led to reduced food insecurity.^20^ However, other studies have shown no association between incentive use and food insecurity.^21^^,^^22^

NI projects have received considerable federal funding over t ime. Authorized in the 2008 Farm Bill, initial federal funds supported the U.S. Department of Agriculture (USDA) Healthy Incentives Pilot to conduct an RCT assessing the impact of incentives for FVs among SNAP participants in Massachusetts.^23^ SNAP participants receiving the incentive increased FVI compared with SNAP participants not receiving the incentive.^24^ Next, the Food Insecurity Nutrition Incentive (FINI) program was funded at $100 million between 2015 and 2018 to facilitate NI projects.^25^ However, the evaluation of the FINI program found no statistically significant change in FVI among participants, although these findings should be interpreted carefully considering study design.^26^ Specifically, the treatment group consisted of participating SNAP households located near FINI redemption sites, although these households may not have been aware of FINI, used the retail outlets that provided FINI, or chose to participate.

**Figure 1:**
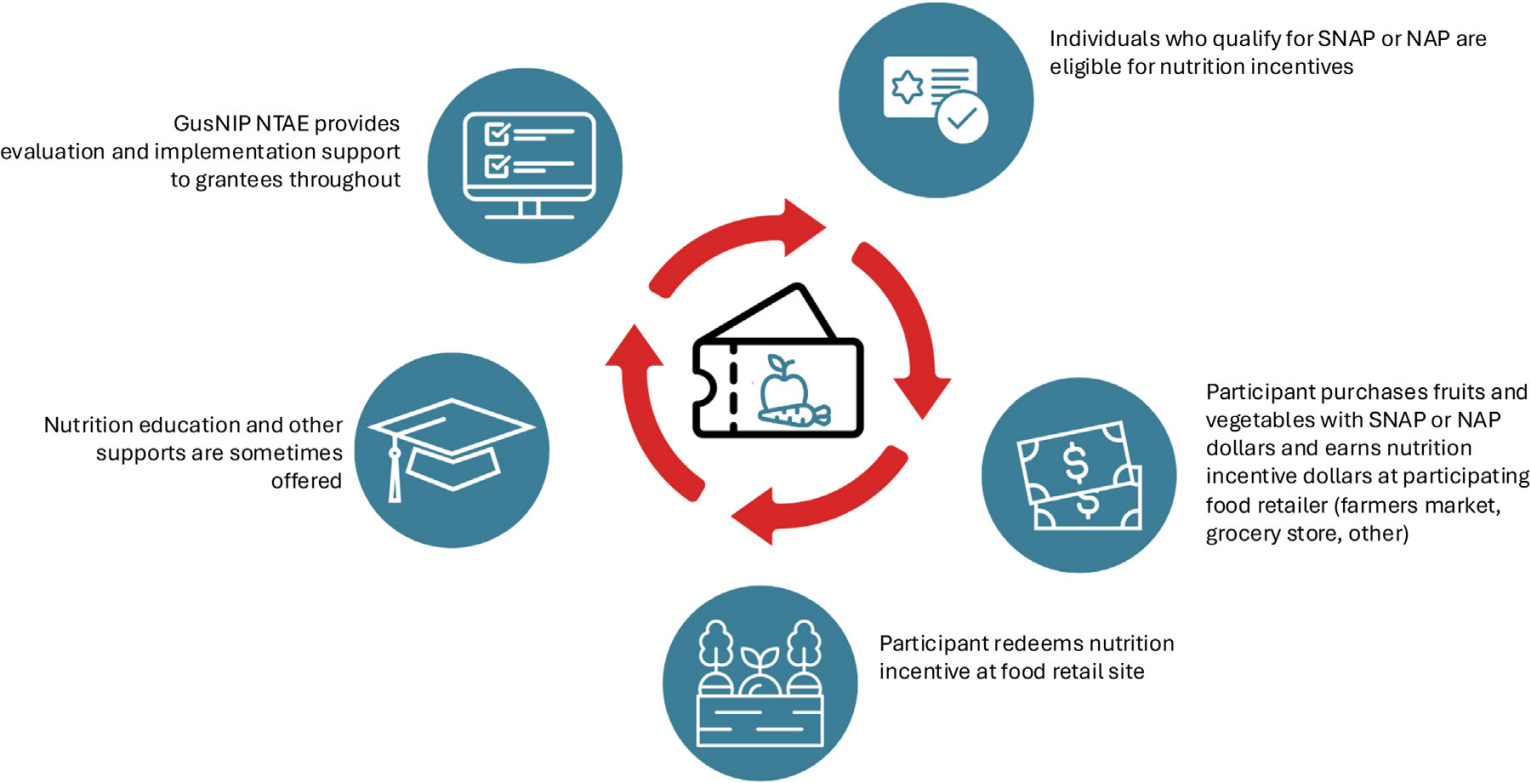
GusNIP model for nutrition incentive program. GusNIP, Gus Schumacher Nutrition Incentive Program; NAP, Nutrition Assistance Program; NTAE, National Training, Technical Assistance, Evaluation, and Information Center; SNAP, Supplemental Nutrition Assistance Program.

The USDA National Institute of Food and Agriculture (NIFA)’s Gus Schumacher Nutrition Incentive Program (GusNIP), appropriated through the 2018 Farm Bill as the successor to the FINI program,^27^ offers competitive grants to support NI and produce prescription projects (the latter not discussed in this paper). Figure 1 describes the general model for GusNIP NI projects. The 2018 Farm Bill also funded the National Training, Technical Assistance, Evaluation, and Information Center (NTAE), which evaluates NI and produce prescription projects.^28^ To understand the associations between NI participation and outcomes, grantees collect survey data from NI participants on the basis of the shared measures survey provided by the NTAE. Then, the NTAE analyzes the data and reports on aggregate outcomes of interest (i.e., FVI, household food insecurity, perceived health).^29^ An opportunity exists to better understand the associations between NI project participation across the U.S. and participant outcomes by examining the large, multiyear NI data set analyzed by the NTAE.

The purpose of this study was to examine the associations between length of NI participation and FVI, food insecurity, and perceived health status in the GusNIP NTAE’s aggregated participant survey data set from 2020–2023. The authors hypothesized that longer participation in NI projects improves FVI, food insecurity, and perceived health.

## METHODS

### Study Population

A cross-sectional design among a convenience sample of NI project participants was used. Data were collected by 65 GusNIP projects (grantees) during their award periods between 2020 and 2023. The research was deemed exempt by the University of Nebraska Medical Center’s IRB (Number 829–20-EX). In this national evaluation, the NTAE had to balance feasibility with rigor and thus selected a cross-sectional design. Longitudinal tracking of participants was not feasible for projects owing to several significant challenges, including the potential for increased stigma associated with SNAP EBT program participation when conducting research in food retail settings that requires matched pre and post data, limited evaluation funding due to grantee budgets that prioritize maximizing NIs for participants, and legal restrictions on monitoring SNAP EBT numbers.

The 65 GusNIP grantees collected anonymous, self-report surveys from a subset of NI project participants annually. All 65 grantees with varying funding cycles are represented in the data set, which included surveys from 8,380 participants across 25 projects in September 2020 to August 2021; 7,072 participants across 50 projects in September 2021 to August 2022; and 8,284 participants across 56 projects in September 2022 to August 2023 (some grantees are represented in multiple years).

The minimum sample size in each study year was based on previous literature to detect 0.25 cups per day difference (Type I error=0.05; power=0.8) in FVI between first-time participants (*n*=860) and participants using an NI project for up to 6 months (*n*=860); participants with >6 months participation were recruited to evaluate the effect of long-term NI participants on FVI.

For all NI projects, eligibility criteria for the study included adult aged ≥18 years, SNAP recipients, and participant of an NI project at the time of the survey. Annually, each GusNIP grantee worked with NTAE staff to identify a sampling plan with a goal to collect 100 or more surveys for each project and implement appropriate and feasible data collection procedures at participating sites. To develop a sampling plan, grantees were asked to define their total population on the basis of eligibility criteria, demographics, and types of food retailers involved. They were also required to determine their sample population and develop a convenience sampling strategy that aimed for representativeness in terms of demographics, types of food retailers, and inclusion of both weekdays and weekend days. The convenience sampling strategy was not based on length of participation to limit bias. Surveys were administered as paper, as digitally based, or over the phone, depending on the sampling plan. A list or count of individuals invited to participate in the survey was not feasible, so the response rate is unknown.

NI projects spanned locations across the U.S. including all 4 NIFA regions (Northeast=23 projects, North Central/Midwest=16 projects, South=12 projects, West=14 projects).^30^ The provision of NIs to SNAP participants for FVs was consistent across implementation. However, NI project implementation varied in terms of the type of food retail (farmers market, grocery store, corner store), the dollar amount of NIs provided (ranging from the most common at 1:1 or 50% off to 2:1–3:1 or 25%–33% off to >75% off), the types of FVs eligible for the incentive (fresh only or fresh, frozen, canned without added sugar or sodium), and the type of incentive instrument used to administer NIs (token, loyalty card, automatic discount).

### Measures

Eligible individuals were invited to complete a cross-sectional survey between 2020 and 2023. The survey included 31 items, with details reported elsewhere.^29^ Participants provided self-reported answers. The survey items analyzed in this study include length of NI participation (first time, ≤6 months, >6 months); FVI; food insecurity; perceived health status (poor, fair, good, very good, and excellent)^31^; sociodemographics (age, sex, race, and ethnicity); and length of SNAP participation (just started, less than a year, more than a year). Specifically, the length of NI participation variable asked, *How long have you been using [Nutrition incentive program name (e.g., Double Up Food Bucks)] to get fruits and vegetables?* FVI was assessed using the 10-Item Dietary Screener Questionnaire Fruit and Vegetable Module, which measures consumption frequency of fruits, fruit juices, and vegetables that results in FV cups per day equivalents.^32^ Food insecurity was measured using the USDA 6-Item Household Food Security Module, which uses a 30-day lookback period to examine food affordability, balanced meals, skipping meals, and reducing meal size.^33^^,^^34^ Notably, first-time participants had no NI influence from the intervention at that time point. Therefore, data on FVI, food security, and perceived health for first-time participants should be interpreted as baseline data, reflecting their status before any intervention exposure. A majority of participants were offered a $10 stipend for their time to complete the survey, although a few projects could not offer the survey stipend owing to budget constraints.

### Statistical Analysis

Statistical analyses examined the associations between the length of NI participation (predictor) and FVI (primary outcome), food security (secondary outcome), and perceived health status (secondary outcome). Length of NI participation (first time, ≤6 months, >6 months) was analyzed as a categorical variable. FVI, a continuous variable, was analyzed by converting Dietary Screener Questionnaire frequency responses into daily frequencies and inputting into a scoring algorithm developed by the National Cancer Institute to estimate daily cup equivalents.^32^ Applying USDA’s scoring protocol, each affirmative response to 6 items about food security received 1 point.^34^ Lower scores identified higher levels of food security (high food security for 0 affirmative responses, marginal food security for 1 affirmative response, low food security for 2–4 affirmative responses, or very low food security for 5–6 affirmative responses). For ease of interpretation, binary analyses were conducted for food insecurity as well as perceived health. The food insecurity variable was simplified by collapsing high and marginal food security into food secure and low or very low food security into food insecure. Perceived health was treated as a binary variable by collapsing good, very good, and excellent versus poor and fair health status.

Unadjusted descriptive statistics were calculated. Linear mixed effects models for FVI and generalized linear mixed-effects models with logit link for food insecurity and perceived health were used to assess the association between length of NI participation and these outcomes; all models accounted for clustering within projects by including a project-specific random effect. Models adjusted for age, sex, race and ethnicity, and length of SNAP participation. Significance level was set at *p*<0.05. All analyses were completed using SAS, Version 9.4 (SAS Institute, Cary, NC).

## RESULTS

Among the 23,736 adults who completed the survey, 4,414 (18.6%) were first-time participants; 7,632 (32.2%) participated ≤6 months; and 11,690 (49.3%) participated >6 months (Table 1). The overall sample was 45.8% (*n*=10,439) non-Hispanic White, 22.0% (*n*=5,002) Hispanic, and 71.9% (*n*=17,517) female. The mean age of the sample was 43.88 (±15.96) years. Participants consumed a mean of 2.74 (±0.8) cups of FVs per day, ranging from 2.64 (±0.9) for first-time participants, to 2.66 (±0.8) for ≤6 months participants, and to 2.84 (±0.8) for >6 months participants. In total, 58.8% (*n*=14,591) were classified as food insecure. Among the total sample, 65.0% (*n*=15,915) reported perceived health status as good, very good, or excellent.

**Table 1:** Characteristics of Participant Sample Stratified by Length of Participation in GusNIP, Descriptive Unadjusted^a^

Characteristic	Total sample	First-time participants	≤6-month participation	>6-month participation
Total, *n* (%)	23,736 (100.0)	4,414 (18.6)	7,632 (32.2)	11,690 (49.3)
Age, years				
18–24	1,712 (7.2)	404 (9.8)	672 (9.4)	513 (4.7)
25–34	6,412 (27.1)	1,037 (25.2)	2,335 (32.6)	2,692 (24.7)
35–44	6,017 (25.4)	896 (21.9)	1,821 (25.4)	2,929 (26.8)
45–64	6,140 (25.9)	1,210 (29.4)	1,639 (22.9)	2,878 (26.7)
≥65	3,419 (14.4)	566 (13.8)	692 (9.7)	1,907 (17.5)
Sex, *n* (%)				
Male	6,268 (25.7)	869 (20.7)	2,035 (27.8)	3,053 (27.1)
Female	17,517 (71.9)	3,245 (77.1)	5,104 (69.8)	7,932 (70.4)
Other	592 (2.4)	93 (2.2)	178 (2.4)	288 (2.6)
Race and ethnicity, *n* (%)				
American Indian or Alaska Native, non-Hispanic	444 (2.0)	71 (2.0)	195 (2.8)	155 (1.5)
Asian, non-Hispanic	1,098 (4.8)	195 (5.4)	349 (4.9)	490 (4.6)
Black or African American, non-Hispanic	4,464 (19.6)	699 (19.3)	1,440 (20.5)	2,146 (20.2)
Native Hawaiian and other Pacific Islander, non-Hispanic	364 (1.6)	22 (0.6)	147 (2.1)	177 (1.7)
White, non-Hispanic	10,439 (45.8)	1,455 (40.2)	3,100 (44.2)	5,159 (48.5)
Other, non-Hispanic	101 (0.4)	15 (0.4)	24 (0.3)	53 (0.5)
More than 1 race, non-Hispanic	861 (3.8)	125 (3.5)	271 (3.9)	418 (3.9)
Hispanic	5,002 (22.0)	1,034 (28.6)	1,487 (21.2)	2,044 (19.2)
Length of SNAP participation, *n* (%)				
Just started	1,691 (7.1)	672 (16.3)	749 (10.2)	206 (1.8)
Less than a year	5,892 (24.9)	866 (21.0)	3,015 (41.1)	1,804 (16.0)
More than a year	16,376 (68.6)	2,588 (62.7)	3,576 (48.7)	9,253 (82.6)
Fruit and vegetable intake, cups per day, mean (SD)	2.74 (0.8)	2.64 (0.9)	2.66 (0.8)	2.84 (0.8)
Food security status, *n* (%)				
Food secure	10,230 (41.2)	1,538 (36.0)	2,993 (40.2)	5,026 (43.9)
Food insecure	14,591 (58.8)	2,730 (64.0)	4,457 (59.8)	6,427 (56.1)
Perceived health, *n* (%)				
Poor or fair	8,560 (35.0)	1,776 (41.7)	2,469 (33.2)	3,740 (32.7)
Good, very good, excellent	15,915 (65.0)	2,481 (58.3)	4,965 (66.8)	7,683 (67.3)

a All *p*<0.0001 for testing the overall difference in these variables among the 3 lengths of NI participation based on chi-square tests. GusNIP, Gus Schumacher Nutrition Incentive Program; NI, nutrition incentive; SNAP, Supplemental Nutrition Assistance Program.

**Figure 2:**
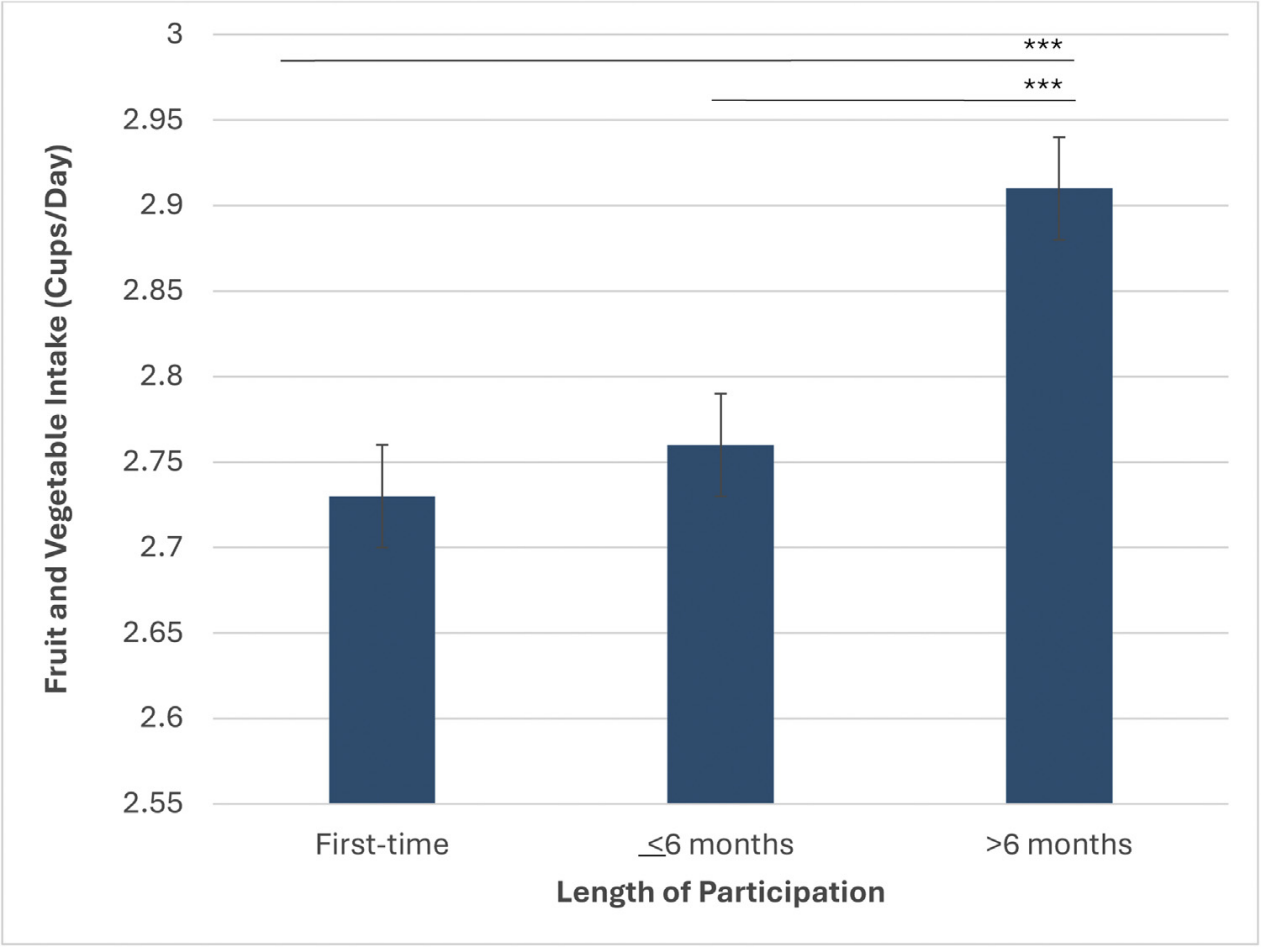
Fruit and vegetable intake by length of participation in GusNIP. *Note:* ***p<0.05. GusNIP, Gus Schumacher Nutrition Incentive Program.

For the multivariate adjusted model on FVI (Figure 2), length of NI participation (*p*<0.0001), age (*p*<0.0001), gender (*p*<0.0001), and race/ethnicity (*p*<0.0001) were statistically significant. Participation in NI at >6 months (cups per day=2.91) demonstrated significantly higher FVI than first-time participation (cups per day=2.73) and participation ≤6 months (cups per day=2.76).

As age increased, FVI also increased, except for those aged ≥65 years (18–24 years=2.72 cups per day, 25–34 years=2.79 cups per day, 35–44 years=2.85 cups per day, 45–64 years=2.89 cups per day, and ≥65 years=2.76 cups per day; *p*<0.0001). Male participants had on average significantly higher FVI than female participants (3.00 vs 2.60 cups per day, *p*<0.0001). Those who identified their sex as other and those who selected more than 1 race reported the highest intake (3.04 and 2.89 cups per day, respectively, *p*<0.0001). The lowest FVI was reported by those who identified as Native Hawaiian and other Pacific Islander (2.67 cups per day, *p*<0.0001) as well as American Indian or Alaska Native (2.63 cups per day, *p*<0.0001).

The multivariate adjusted model for food insecurity (Table 2) was associated with length of NI participation (*p*<0.0001), age (*p*<0.0001), race or ethnicity (*p*<0.0001), and length of SNAP participation (*p*<0.0001). The odds of food insecurity were 34% lower among ≤6-month group than among the first-time participants (OR=0.66, 95% CI=0.60, 0.73) and 39% lower among >6-month group than among the first-time participants (OR=0.61, 95% CI=0.56, 0.67). All other age groups had higher odds of experiencing food insecurity than people who were aged ≥65 years (ORs range from 1.47 to 1.76). Other racial groups experienced higher odds of food insecurity, including American Indian or Alaska Native participants (OR=1.74, 95% CI=1.37, 2.22), Native Hawaiian and other Pacific Islander participants (OR=1.57, 95% CI=1.19, 2.08), Black participants (OR=1.19, 95% CI=1.08, 1.30), and Hispanic participants (OR=1.47, 95% CI=1.34, 1.61), than non-Hispanic White participants. Participants who received SNAP benefits for less than a year had significantly lower odds of experiencing food insecurity than individuals who just started (OR=0.81, 95% CI=0.70, 0.92).

**Table 2:** Logistic Regression for Food Insecurity and Perceived Health by Length of Participation in GusNIP, Multivariate Adjusted Model

Characteristic	Food insecurity		Perceived general health	
	OR	95% CI	OR	95% CI
Total				
First-time participants	ref	ref	ref	ref
<6-month participation	0.66	0.60, 0.73	1.21	1.10, 1.33
>6-month participation	0.61	0.56, 0.67	1.34	1.23, 1.47
Age, years				
18–24	1.49	1.29, 1.73	2.28	1.95, 2.67
25–34	1.47	1.33, 1.63	2.04	1.84, 2.26
35–44	1.52	1.37, 1.67	1.61	1.46, 1.77
45–64	1.76	1.60, 1.93	0.94	0.86, 1.03
≥65	ref		ref	
Sex				
Male	1.01	0.94, 1.09	1.11	1.03, 1.20
Female	ref		ref	
Race and ethnicity				
American Indian or Alaska Native, non-Hispanic	1.74	1.37, 2.22	0.81	0.64, 1.01
Asian, non-Hispanic	0.79	0.69, 0.92	0.88	0.76, 1.02
Black or African American, non-Hispanic	1.19	1.08, 1.30	1.12	1.02, 1.23
Native Hawaiian and other Pacific Islander, non-Hispanic	1.57	1.19, 2.08	0.63	0.49, 0.81
White, non-Hispanic	ref	ref	ref	ref
Other, non-Hispanic	1.21	0.79, 1.85	1.78	1.02, 3.12
More than 1 race, non-Hispanic	1.16	1.00, 1.35	0.98	0.84, 1.14
Hispanic	1.47	1.34, 1.61	0.78	0.72, 0.86
Length of SNAP participation				
Just started	ref	ref	ref	ref
Less than a year	0.81	0.70, 0.92	1.09	0.95, 1.26
More than a year	1.03	0.90, 1.17	0.82	0.72, 0.93

GusNIP, Gus Schumacher Nutrition Incentive Program; SNAP, Supplemental Nutrition Assistance Program.

For perceived health (Table 2), significant effects in the multivariate adjusted model included length of NI participation (*p*<0.0001), age (*p*<0.0001), gender (*p*<0.0001), race or ethnicity (*p*<0.0001), and length of SNAP participation (*p*<0.0001). Participants in both ≤6-month and >6-month groups were more likely to report better perceived health than first-time participants (OR=1.21, 95% CI=1.10, 1.33 and OR=1.35, 95% CI=1.23, 1.47). Age groups of 18–24 years, 25–34 years, and 35–44 years experienced higher odds of better health than people aged ≥65 years (OR=2.28, 95% CI=1.95, 2.67; OR=2.04, 95% CI=1.84, 2.26; and OR=1.61, 95% CI=1.46, 1.77). In addition, Native Hawaiian and other Pacific Islander participants (OR=0.63, 95% CI=0.49, 0.81) and Hispanic participants (OR=0.78, 95% CI=0.72, 0.86) were less likely to report better health than non-Hispanic White participants. Participants who reported using SNAP benefits for more than a year had significantly lower odds of reporting better health than those who just started to use the benefit (OR=0.82, 95% CI=0.72, 0.93).

## DISCUSSION

In a nationwide cross-sectional cohort of 65 GusNIP-funded grantees and 23,726 survey participants, longer participation in NI projects was associated with significantly greater FVI, food security, and perceived health. Previous studies evaluating effects of NI projects have reported varying outcomes.^11^, ^12^, ^13^, ^14^, ^15^, ^16^^,^^20^, ^21^, ^22^ In this study, the authors found a positive relationship between length of NI participation and FVI and perceived health as well as an inverse relationship between length of NI participation and food insecurity. These findings suggest that NIs distributed through GusNIP may be a promising strategy to support FV intake, food security, and overall health among SNAP participants.

A primary goal of GusNIP NI projects is to increase FVI among SNAP participants. The 2020 U.S. Dietary Guidelines for Americans recommends consuming 3.5–5 cups of FVs per day (or 2–3 cups of vegetables and 1.5–2 cups of fruit per day).^1^ Participants of any length of NI participation consumed a mean of 2.74 cups of FVs per day, which is greater than the national average of 2.58 cups of FVs per day.^35^ After longer NI participation, this increased to 2.84 cups of FVs per day but still fell short of U.S. Dietary Guidelines for Americans recommendations.^1^ These findings are encouraging because prior research demonstrates a dose–response relationship between FVI and health, including decreased mortality risk.^36^

The study findings demonstrate that significant differences in FVI exist by racial and ethnic groups, which is also observed in the U.S. population.^2^ Intentional efforts are needed to address differences in outcomes between racial and ethnic groups, including among NI projects. For instance, some studies have noted the effectiveness of providing culturally appropriate foods, education, and/or tailored evaluation tools when working with SNAP participants.^37^^,^^38^

As age increased, FVI also increased, except for those aged ≥65 years. FVI was lowest overall for those aged 18–24 years, which is a trend mirrored in the U.S. population.^2^ These findings suggest that NI projects should be tailored to specific age groups, exploring factors that may be unique to these populations such as digestion considerations or convenience, desirability, and palatability of FVs.

Food security status improved with longer participation in NI projects, which is important given that the rate of food security is substantially lower in the study population (41.2% food secure) than a national average of 87.2% in 2022.^39^ This lower rate of food security could also demonstrate that NI programs reach populations who need it most. Compared with food insecurity among individuals aged ≥65 years, food insecurity was elevated among all other age groups. This finding may be due in part to younger adults being more likely to have children (than older adults) and that FVs received through NI projects may be more spread out, meaning that a set amount of FVs is shared among all the people in the household. In addition, households with children experience food insecurity at rates higher than the national average.^40^ Food insecurity may be difficult to self-report, so it is important to consider that these values may be underreported.

Approximately 35.0% of study participants reported fair or poor health. In comparison, 11.2% of the U.S. population indicated fair or poor health as reported by the National Health Interview Survey during 2019.^41^ Moreover, 2019 national sample data showed that 24.6% living below the federal poverty line reported fair or poor health.^42^ These findings show that perceived health improved with longer participation in NI projects. Although subjective in nature, this single-item measure of perceived health is well established as a predictor of actual health, including morbidity and mortality associated with diet-related chronic conditions.^43^, ^44^, ^45^ As such, the results point to longer participation in NI projects being protective against the types of poor health outcomes that populations with low income face.

Study participants reported characteristics similar to those of national SNAP participants.^46^ In this study, the authors assessed length of SNAP participation because the program is intended to influence food choices, food security, and health outcomes.^47^ The authors found that individuals participating in SNAP for less than a year had lower odds of food insecurity, whereas SNAP participants for more than a year did not. SNAP benefits can provide immediate relief from food insecurity, although prolonged SNAP participation may indicate underlying, persistent poverty that SNAP alone may not be able to address. Furthermore, individuals who reported SNAP participation for more than a year had lower odds of reporting better health than those who had just started SNAP. Extended SNAP participation may reflect deeper challenges that lead to a lack of access to resources, such as health care, which can negatively affect health outcomes. Study data show that NI projects may offer strategies to support food security and perceived health, and future research should explore the underlying mechanisms and assess whether these associations are sustained over time and for which segments of the population.

### Limitations

Because NI projects usually reach a large sample, the NTAE’s evaluation design is cross-sectional and focuses on associations related to desired outcomes. Although cross-sectional designs are feasible for aggregate and large-scale evaluation, there are limitations for evaluating project impact over time. Longitudinal, randomized, or matched-controlled studies would provide a more internally valid means to determine causality, but this was not possible per this funding mechanism and NI project implementation, on a national scale. NI projects do not have the capability to match participant data nor a large budget to dedicate to evaluation, and these projects face restrictions with accessing SNAP EBT numbers. Because GusNIP grantees have flexibility in implementation, future research should explore key NI participant outcomes in comparison with heterogenous project models in type of food retail, the dollar amount of NI provided, the types of FVs eligible for the incentive, and the type of incentive instrument used for FVs. Furthermore, the degree of involvement in the length of participation variable may have varied among participants. For instance, a participant with >6 months of involvement may have spent their NI regularly, once a week, or only sporadically a few times over the past several months. Thus, future research should consider exploring dosage variations within the different participation durations. Directionality or order of outcomes was not assessed (e.g., which outcome program participation improves first). For instance, it is unknown whether FVI improved before or after food insecurity.

## CONCLUSIONS

USDA NIFA’s GusNIP provides a momentous opportunity to understand the associations between national Farm Bill policy and public health outcomes. Although grantees implemented and collected data about their unique NI projects, the GusNIP NTAE was able to evaluate the public health–associated outcomes of NIs on a national scale. In a large cross-sectional sample, NIs across multiple GusNIP projects had a positive associated effect on FVI, food security, and perceived health of participants across multiple years, across thousands of participants, and in various communities by length of participation.
